# Adherence to ESGE guidelines on biliary stenting in malignant distal strictures: Results from a prospective Italian registry

**DOI:** 10.1055/a-2777-9199

**Published:** 2026-01-26

**Authors:** Tommaso Schepis, Rocco Maurizio Zagari, Stefano Francesco Crinó, Marco Sacco, Enrico Palmeri, Roberto Grassia, Alessio Santagati, Giovanna Venezia, Nicola Olivari, Alba Panarese, Massimiliano Mutignani, Ivano Biviano, Helga Bertani, Massimo Devani, Samuele de Minicis, Giuseppe de Roberto, Antonio Aucello, Socrate Pallio, Armando Gabbrielli, Sebastian Manuel Milluzzo, Maria Caterina Parodi, Luigi Pasquale, Guido Costamagna, Elton Dajti, Andrea Tringali, and the PROTESIED Study Group, Claudio Giovanni De Angelis, Biagio Elvo, Alessandro Risso, Marianna Bravo, Raffaele Macchiarelli, Silvia Cocca, Gianpiero Manes, Giampiero Macarri, Pietro Soru, Simonetta Rapacchietta, Melita Giuseppinella

**Affiliations:** 1 Digestive Endoscopy Unit, Fondazione Policlinico Gemelli IRCCS – Catholic University, Rome, Italy; 2 9296 Department of Medical and Surgical Sciences, University of Bologna, Bologna, Italy; 3 19051 Pancreatic Diagnostic and Interventional Endoscopy Unit, University of Verona, Verona, Italy; 4 Endoscopy Unit, Gastroenterology Department, Azienda Ospedaliero-Universitaria Città della Salute e della Scienza di Torino, Torino, Italy; 5 Pancreatic Diagnostic and Interventional Endoscopy Unit, University of Verona, Verona, Italy; 6 UOSD Endoscopia Digestiva e Gastroenterologia, ASST di Cremona, Cremona, Italy; 7 Dipartimento di Medicina, UOSD Gastroenterologia ed Endoscopia, P. O. G. Fogliani Milazzo, Milazzo, Italy; 8 208961 U.O.C of Gastroenterology, Ospedale Santa Croce, Cuneo, Italy; 9 Digestive Endoscopy Unit and Gastroenterology, Fondazione Poliambulanza, Brescia, Italy; 10 Division of Gastroenterology and Digestive Endoscopy, Department of Medical Sciences, Central Hospital-Azienda Ospedaliera, University “Aldo Moro” of Bari, Taranto, Italy; 11 Endoscopia Digestiva ed Interventistica, ASST Grande Ospedale Metropolitano Niguarda, Milano, Italy; 12 Gastroenterology and Interventional Endoscopy Unit, AOUS Policlinico S. Maria alle Scotte, Siena, Italy; 13 Gastroenterologia ed Endoscopia Digestiva, Azienda Ospedaliero Universitaria Policlinico di Modena, Modena, Italy; 14 472771 Division of Gastroenterology and Digestive Endoscopy, ASST Rhodense, Rho, Italy; 15 Gastroenterologia ed Endoscopia Digestiva Ospedale, Ospedale A. Murri, AST Fermo, Fermo, Italy; 16 Division of Endoscopy, European Institute of Oncology, Milan, Italy; 17 UO Endoscopia Digestiva, Ospedale “M.G. Vannini”, Rome, Italy; 18 Endoscopy Unit, G Martino University Hospital of Messina, Messina, Italy; 19 UO Multizonale di Gastroenterologia ed Endoscopia Digestiva, APSS Ospedale Santa Chiara, Trento, Italy; 20 Digestive Endoscopy Unit and Gastroenterology, Fondazione Poliambulanza, Brescia, Italy; 21 IRCCS University Hospital San Martino-IST National Institute for Cancer Research, Gastroenterology and Digestive Endoscopic Unit, Genova, Italy; 22 UOC Gastroenterologia ed Endoscopia Digestiva, ASL Avellino, Italy; 23 Digestive Endoscopy Unit, Catholic University, Rome, Italy; 24 Endoscopy Unit, Ospedale Isola Tiberina - Gemelli Isola, Rome, Italy; 25 Centre for Endoscopic Research, Therapeutics and Training (CERTT), Rome, Italy

**Keywords:** Pancreatobiliary (ERCP/PTCD), Strictures, ERC topics, Tissue diagnosis

## Abstract

**Background and study aims:**

Distal malignant biliary strictures (dMBSs) are a common indication for endoscopic retrograde cholangiopancreatography (ERCP). The present study aimed to evaluate adherence of Italian endoscopic centers to European Society of Gastrointestinal Endoscopy (ESGE) guidelines on management of dMBS.

**Patients and methods:**

This prospective cohort, observational, multicenter study was promoted by the Italian Society of Digestive Endoscopy. All consecutive patients with dMBS were included in the registry. Clinical and technical data were recorded. Clinical follow-up was performed at 7 and 30 days, and then every 3 months. Adherence to the eight ESGE recommendations (defined as full-, intermediate- and poor-adherence if > 85%, ≥ 65% to ≤85%, and < 65%, respectively) was considered the primary outcome.

**Results:**

Seventeen Italian endoscopy centers were included. Between January 2020 and January 2022, 827 patients were included. Full adherence to the guidelines was reported for post-ERCP acute pancreatitis prophylaxis, retreatments, and preoperative biliary drainage. Intermediate adherence was reported for type of stent used in palliative drainage (85% SEMS and 15% plastic stents). Poor adherence was reported for type of stent used in preoperative drainage (56% self-expandable metal stents [SEMSs]), availability of pathological diagnosis in case of U-SEMS placement (45% of U-SEMSs placed without pathologically diagnosis), antibiotic prophylaxis (70.6%), and sphincterotomy (88%).

**Conclusions:**

Adherence to ESGE guidelines needs to be improved in specific areas, including excessive use of plastic stents, use of U-SEMS without pathological diagnosis, and routine performance of sphincterotomy and use of antibiotic prophylaxis. (ClinicalTrials.gov ID: NCT05761496)

## Background and study aims

Distal malignant biliary stricture (dMBS) is often seen in various cancers, particularly pancreatic cancer 1 2 3 . Endoscopic retrograde cholangiopancreatography (ERCP) is the preferred method for biliary drainage in these patients, with plastic stents (PSs) and self-expandable metal stents (SEMSs) being used 4 5 . PSs are easily removable but prone to migration and early occlusion 6 7 . SEMSs are available in three types: uncovered (U-SEMS), partially-covered (PC-SEMS), and fully-covered (FC-SEMS) 8 . U-SEMSs have low migration risk but higher risk of tissue ingrowth impeding their removability, whereas PC-SEMSs and FC-SEMSs are removable but risk of migration is increased 9 . Moreover, controversial results regarding risk of post-ERCP cholecystitis due to cystic duct occlusion by covered SEMSs have been published 10 11 12 .

In 2018, the European Society of Gastrointestinal Endoscopy (ESGE) published updated guidelines on endoscopic biliary stenting. The following list summarizes the statements regarding dMBS 13 . In summary, ESGE recommended use of SEMSs to treat dMBS in both preoperative and palliative settings 13 , without performing routine biliary sphincterotomy and avoiding U-SEMS if a diagnosis of malignancy was not yet obtained. These recommendations are based on the clear advantage of SEMSs over PSs (lower rate of endoscopic reintervention, longer patient survival, lower risk of stent dysfunction/cholangitis) 14 15 . However, the choice among the different types of SEMSs is still debated. A recent meta-analysis, including 13 studies (7 randomized controlled trials [RCTs]) and 2,239 patients, showed no statistically significant difference in the survival benefit and overall adverse event (AE) rate between U-SEMSs and FC-SEMSs 16 . Performing sphincterotomy was not recommended due to a higher risk of bleeding without a protective effect on post-ERCP pancreatitis, as demonstrated by a recent meta-analysis 17 . Despite the better outcome associated with SEMSs in dMBS when compared with PSs, in real-life experience, SEMS patency is 4 to 6 months 18 19 . SEMS occlusion is associated with the worst oncological outcome, because patients develop cholangitis requiring chemotherapy withdrawal. In pancreatic cancer, SEMS malfunction is associated with shorter overall survival and progression-free survival 18 ; therefore, it is crucial to choose the correct stent for each patient to allow optimal biliary drainage.

### European Society of Digestive Endoscopy (ESGE) statements for endoscopic drainage of distal malignant biliary stricture 13

- ESGE recommends against routine preoperative biliary drainage in patients with malignant extrahepatic biliary obstruction; preoperative biliary drainage should be reserved for patients with cholangitis, severe symptomatic jaundice (e.g. intense pruritus) or delayed surgery or before neoadjuvant chemotherapy in jaundiced patients.
- ESGE recommends the endoscopic placement of a 10-mm diameter self-expandable metal stent (SEMS) for the preoperative biliary drainage of extrahepatic malignant biliary obstruction.
- ESGE recommends SEMS insertion for the palliative drainage of malignant extrahepatic biliary obstruction.
- ESGE recommends against the insertion of U-SEMS for the drainage of extrahepatic biliary obstruction of an unconfirmed etiology.
- ESGE suggests that in a patient with a distal malignant biliary stricture and a non-functioning stent, a plastic stent should be replaced by a SEMS and, in the case of a SEMS, a plastic stent or a new SEMS should be inserted within the original SEMS.
- ESGE recommends, for the prophylaxis of post-ERCP pancreatitis, the routine administration of 100 mg of diclofenac or indomethacin intrarectally immediately before or immediately after ERCP in every patient with no contraindication.
- ESGE suggests the administration of antibiotic prophylaxis before biliary stenting in selected patients (e. g., immunocompromised patients, those expected to have incomplete biliary drainage).
- ESGE suggests against routine endoscopic biliary sphincterotomy before inserting a single plastic or an U/PC SEMS.

The present study aimed to create an Italian national multicenter prospective registry to evaluate real-life adherence of endoscopists to ESGE guidelines on endoscopic management of dMBS.

## Patients and methods

### Study design

The present study was a prospective, observational, multicenter study. The Italian Society of Digestive Endoscopy (SIED) invited all their members to participate to the present study (PROTESIED Study).

### Patient population

All patients with biliary obstruction secondary to malignant stenosis of the mid-distal common bile duct were eligible to be included in the study, as part of the normal care pathway. Inclusion criteria were as follows: age > 18 years, radiological diagnosis of neoplastic biliary stricture, first endoscopic treatment (naïve papilla), and patients candidates for both palliative and preoperative biliary drainage. Exclusion criteria were as follows: previous endoscopic or percutaneous biliary drainage, stricture located < 3 cm from the main hepatic confluence (measured during fluoroscopy considering that the duodenoscope has a diameter of approximately 13 mm), neoplastic duodenal stenosis/infiltration with inaccessible papilla, altered anatomy, concomitant treatment with biliary radiofrequency or other endoluminal therapy, final diagnosis of nonneoplastic pathology (e.g. chronic/autoimmune pancreatitis), pregnant or breastfeeding women, and patients in emergency situations.

Patients who met the inclusion criteria were carefully informed about the study and provided a dedicated informed consent.

### Clinical follow-up

Patients were contacted by telephone after 7 and 30 days and every 3 months to verify absence of cholangitis and possible need for endoscopic reintervention and to evaluate the progress of any chemoradiation treatments undertaken after biliary drainage. Follow-up was interrupted after 12 months. Follow-up was interrupted at time of first episode of cholangitis (considered a sign of stent misfunction, the endoscopic treatment was recorded), surgical resection, or death.

### Data collection

The following data were collected for each patient: demographics; types and neoplasm stage; liver function tests (LFTs) before drainage (bilirubin, alanine transaminase, alkaline phosphatase); gallbladder status (alithiasic, lithiasic or previous cholecystectomy); histology at time of drainage (available and not available ); ERCP success (defined as successful biliary cannulation and stent placement); biliary sphincterotomy (performed or not); type and characteristics (length and diameter) of the implanted stent; if the gallbladder was in place, involvement of the cystic duct by the stricture was annotated during cholangiogram (yes, no, cystic duct not opacified); purpose of biliary drainage (preoperative, palliative, prechemotherapy); data relating to any surgical intervention (type of surgery, occurrence of cholangitis before surgery, surgical problems related to presence of the stent, LFTs before surgery); and AEs (pancreatitis, cholangitis, cholecystitis, bleeding, perforation) and severity (mild, moderate, severe), which were defined according to ESGE criteria 20 .

### Statistical analysis

Continuous variables are described by means and standard deviation and compared using student *t* -test or analysis of variance. Discrete variables are expressed as percentage with a 95% confidence interval (CI) and compared using Chi-square test.

Incidence of complications was calculated for each type of biliary stent and is expressed as incidence rate. The association between patient baseline characteristics, type of stent used, and development of complications was evaluated by calculating relative risk using a univariate regression analysis or, if possible, a multivariable logistic regression analysis adjusted for sex and age. *P* < 0.05 was considered statistically significant. Statistical analyses were conducted anonymously and carried out using STATA (STATA Corp., College Station, Texas, United States).

### Study outcomes

The primary outcome was adherence of Italian endoscopists to the eight ESGE-guideline recommendations on endoscopic drainage of dMBS, including a total of 10 issues. Adherence to the eight ESGE recommendations was arbitrarily defined as “full”, “intermediate” and “poor” if > 85%, ≥ 65% to ≤ 85%, and < 65% of patients were treated according to the ESGE recommendation, respectively.

## Results

### General data

**Fig. 1:**
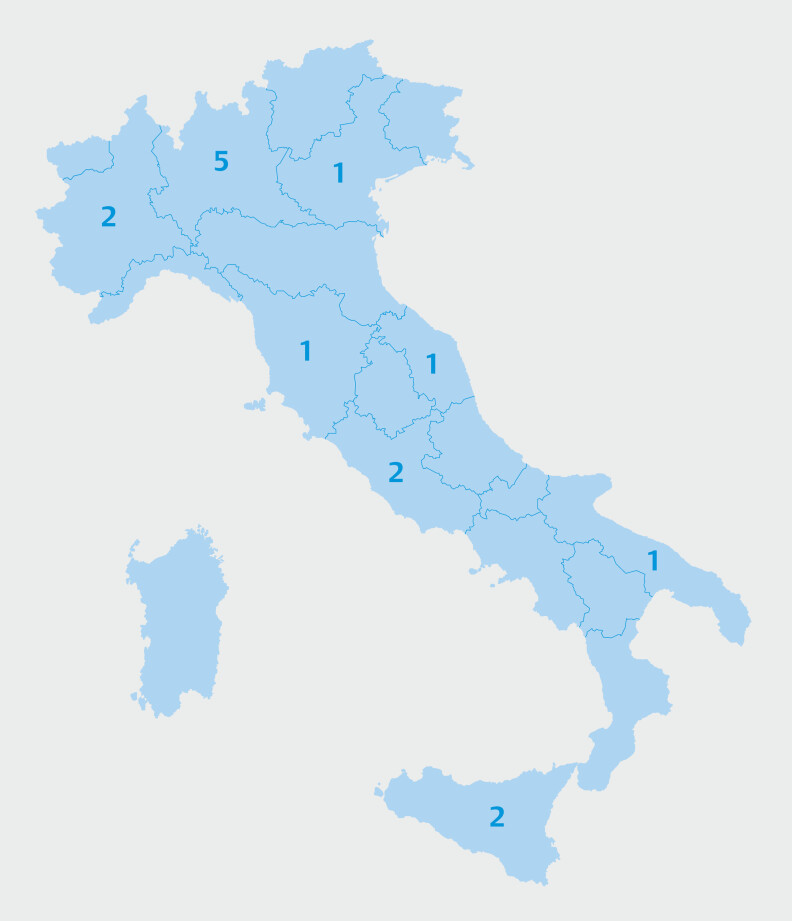
Italian centers involved in the PROTESIED registry.

Seventeen Italian centers adhered to the study (10 from the north, 4 from central Italy, and 3 from southern Italy) ( Fig. 1 ). Between January 2020 and January 2022, 871 patients were included. All of the participating centers obtained local Ethics Committee approval for the study.

Ten patients were excluded due to incomplete data collection and 16 due to ERCP failure (10 cases of duodenal stricture that prevented the stent from reaching the papilla, six cases of difficult papillary cannulation). Therefore, a total of 845 patients were included in the final analysis ( Table 1 ). Eighteen patients did not have any follow-up data, so they were excluded from the longitudinal cohort. Of the remaining 827 patients, 142 (17%) completed 12-month follow-up, 177 (21.4%) underwent surgery, 245 (29.6%) died during follow-up, 165 (19.9%) developed cholangitis, and 98 (11%) were lost to follow-up.

**Table:** **Table 1** Patient and center characteristics.

Variable	Overall population (n = 845)
Age (SD)	71 (12)
	N (%)
Sex (male)	433 (51.6)
Geographic area of the Italian endoscopy center	N (%)
North	513 (60.7)
Centre	224 (26.5)
South	108 (12.8)
University hospital	470 (55.6)
SD, standard deviation.	

In the majority of cases, dMBS etiology was pancreatic head cancer (79.3%) ( Table 2 ) *.* In 65.4% of patients, a pathological diagnosis was not available at time of ERCP.

**Table:** **Table 2** Neoplasia characteristics.

Variable	All patients (n = 845)
Etiology of malignant biliary obstruction	N (%)
Pancreatic cancer	664 (78.5)
Distal cholangiocarcinoma	92 (10.9)
Ampullary cancer	48 (5.7)
Metastases	26 (3.1)
Other	15 (1.8)
Disease stage	N (%)
Resectable	265 (31.4)
Locally advanced	350 (41.4)
Metastatic disease	230 (27.2)
Gallbladder stones (missing data n = 5)	N (%)
No	604 (71.9)
Yes	134 (16)
Previous cholecystectomy	102 (12.1)
**Laboratory tests**	Mean (range)
Bilirubin levels (mg/dL)	13 (6.9)
ALT (IU/L)	204 (108–329)
γ-GT (IU/L)	510 (310–854)
ALP (IU/L)	412 (284–632)
ALP, alkaline phosphatase; ALT, alanine transaminase; γ-GT, gamma glutamyl transferase.	

SEMS was the most common type of biliary stent used (77.6%). ERCP technical details are summarized in Supplementary Table 1.

In multivariate analysis, patient characteristics were compared among the different geographical areas (north, central and southern Italy) (Supplementary Table 2). Histopathological diagnosis was more frequently available at time of ERCP in centers from southern Italy ( *P* = 0.0001). SEMSs were more commonly used in centers from northern Italy ( *P* = 0.0001), whereas no differences were found in terms of the demographics, stricture etiology, or disease stage among the centers.

### Adherence to guidelines

**Fig. 2:**
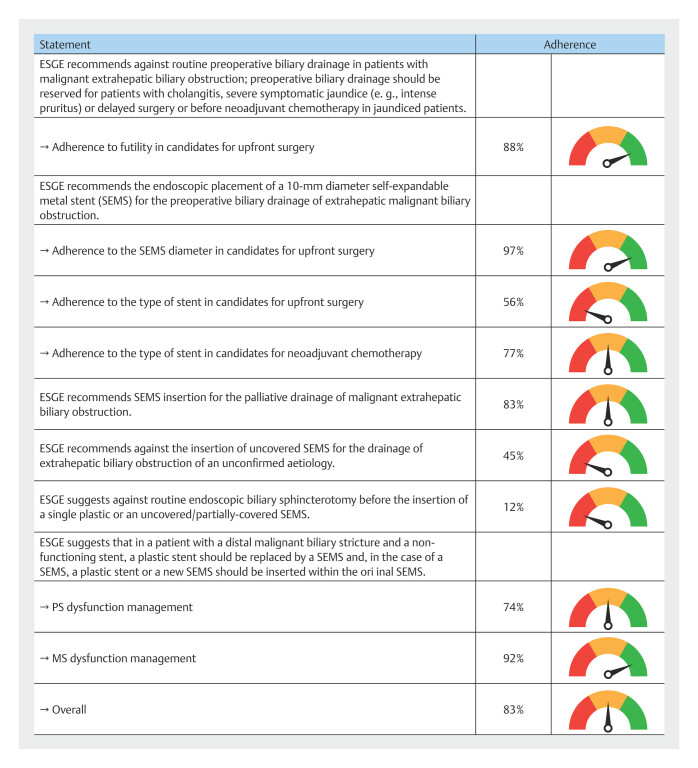
Overall adherence to the ESGE guidelines 13 .

Fig. 2 summarizes overall adherence to the ESGE guidelines.

ESGE recommends against routine preoperative biliary drainage in patients with malignant extrahepatic biliary obstruction; preoperative biliary drainage should be reserved for patients with cholangitis, severe symptomatic jaundice (e.g. intense pruritus) or delayed surgery or before neoadjuvant chemotherapy in jaundiced patients.

Among the operable patients, 180 underwent pancreatic surgery with curative intent. In 78% of cases, the biliary drainage was effective, and surgery was performed without any episodes of cholangitis. In 22% of operable patients, cholangitis due to stent obstruction occurred before surgery.

Altogether, 177 patients were candidates for preoperative biliary drainage; 71% of those undergoing preoperative biliary drainage had a bilirubin level >10 mg/dL and 88% of patients had surgery >7 days after biliary drainage with an adequate time frame.

ESGE recommends endoscopic placement of a 10-mm-diameter SEMS for preoperative biliary drainage of extrahepatic malignant biliary obstruction.

SEMSs were placed in 59.2% of cases (10 U-SEMS, 3 PC-SEMS, and 64 FC-SEMS) and 40.8% received a plastic stent. Among the patients receiving metal stents, 97.4% had a 10-mm SEMS.

ESGE recommends SEMS insertion for palliative drainage of malignant extrahepatic biliary obstruction.

A total of 467 patients were candidates for palliative biliary drainage, 85% of whom received a SEMS and 15% a plastic stent. In multivariate analysis, use of PSs was associated with university hospital, biliary tract cancer, locally advanced cancer, resectable cancer, histopathology not available at time of ERCP, and the patient being a candidate for chemotherapy ( **Supplementary Table 3** ).

ESGE recommends against insertion of U-SEMS for drainage of extrahepatic biliary obstruction of an unconfirmed etiology.

Altogether, 662 SEMSs were placed, with 178 (27.3%) being U-SEMS; 55% of patients who received a U-SEMS did not have a pathology-confirmed diagnosis at time of ERCP and 91.6% of these patients had a final histology positive for malignancy, whereas 15 patients (8.4%) did not receive biopsy and died due to age and comorbidity precluding any treatment.

ESGE suggests that in a patient with a distal malignant biliary stricture and a non-functioning stent, a plastic stent should be replaced by a SEMS and, in the case of a SEMS, a plastic stent or a new SEMS should be inserted within the original SEMS.

During follow-up, 75 patients (8.9%) received a second ERCP for stent dysfunction. Of them, 38 had a plastic stent, whereas 37 had a SEMS; 92% of the patients with occluded SEMSs were treated with a new SEMS inside the previous one, whereas the remaining 8% had a plastic stent inserted into the SEMS. Occluded PSs were managed by plastic stent exchange (26%) and SEMS placement (74%).

ESGE recommends, for the prophylaxis of post-ERCP pancreatitis, routine administration of 100 mg of diclofenac or indomethacin intrarectally immediately before or immediately after ERCP in every patient with no contraindication.

Routine rectal administration of 100 mg indomethacin was reported by 94.1% of centers.

ESGE suggests administration of antibiotic prophylaxis before biliary stenting in selected patients (e. g., immunocompromised patients, those expected to have incomplete biliary drainage).

In 29.4% of centers, antibiotic prophylaxis was administered in selected cases; however, 70.6% of centers administrated antibiotic prophylaxis in all patients undergoing ERCP without distinctions.

ESGE recommends against routine endoscopic biliary sphincterotomy before inserting a single plastic or a U/PC-SEMS.

Biliary sphincterotomy was performed in 87.9% of patients undergoing biliary drainage. No differences were reported in sphincterotomy performance between patients receiving plastic stent, U-SEMS, or PC-SEMS (87.4%) and those receiving FC-SEMS (87.5%).

Moreover, a subgroup analysis was performed to evaluate any statistically significant difference in ESGE guideline adherence among the different Italian geographic areas ( **Supplementary Table 4** ) and hospital settings (university hospital vs. community hospital) ( **Supplementary Table 5** ). Use of SEMSs in the preoperative setting was significantly lower in central and southern Italy, whereas use of SEMSs in patients receiving neoadjuvant chemotherapy was significantly higher in central Italy. Nevertheless, the central areas were more commonly associated with U-SEMS placement in patients without a confirmed pathological diagnosis. Regarding hospital setting, U-SEMS placement in patients without a confirmed pathological diagnosis was more common in university hospitals, whereas endoscopic sphincterotomy was more commonly performed in non-university hospitals.

### Adverse events

Early AEs occurred in 10.7% of patients within 7 days post-endoscopic procedure, including acute pancreatitis, which was always managed conservatively, although two patients died during hospitalization. Sphincterotomy bleeding was typically managed endoscopically, with one patient requiring surgery due to neoplastic infiltration for endoscopic treatment failure. No bleeding-related mortality was reported. Cholangitis was treated with ERCP in 81% of patients, with no related mortality and was managed in all patients with antibiotics. Cholecystitis was rare, with four patients managed with antibiotics (n = 2), by percutaneous drainage (n = 1), and cholecystectomy (n = 1).

Intermediate AEs (between 8 and 30 days) were reported in 42 patients (5.1%). Among the patients with cholangitis, 75% underwent a second ERCP, 21% were managed conservatively with antibiotics, and 4% received a percutaneous transhepatic drainage; 55.2% of the patients with cholangitis were previously drained with PSs and the cause of plastic stent dysfunction was occlusion in 71.4% and distal migration in 28.6% of cases. Among the patients with SEMSs who developed cholangitis (44.8%), mechanisms of dysfunction were SEMS occlusion by biliary sludge (30.8%), distal stent migration (30.8%), proximal stent migration (23.1%), and tumor ingrowth (7.7%).

Among patients who developed intermediate AEs, 9.7% required a chemotherapy or radiation therapy withdrawal to allow management of the complication.

Late AEs (after 30 days) were reported in 81 patients (10.6%). Among patients with cholangitis, 79% underwent a second ERCP, 20% were managed conservatively with antibiotics, and1% received percutaneous transhepatic drainage; 36.2% of patients with cholangitis were previously drained with PSs and the cause of plastic stent dysfunction was occlusion in 68%, distal migration in 24%, and proximal migration in 12% of patients.

Among patients with SEMS who developed cholangitis, mechanisms of dysfunction were SEMS occlusion by biliary sludge (39.5%), distal stent migration (18.4%), proximal stent migration (5.3%), and tumor ingrowth (34.2%). Patients with cholecystitis were treated conservatively with antibiotics in 50% of cases; cholecystectomy was required in two cases. Patients with liver abscesses were treated with antibiotics in all cases, but one also required percutaneous drainage.

## Discussion

Distal malignant biliary obstruction is a common scenario for the biliopancreatic endoscopist. ERCP is still considered the gold standard treatment, and it has a lower AE rate compared with percutaneous drainage 21 22 23 . ERCP is highly effective, and in our national registry, ERCP failure was reported only in six of 871 cases (0.6%). The very low rate of cannulation failure in our series can be secondary to the diffusion of “advanced” cannulation techniques (double guidewire, transpancreatic sphincterotomy, precut) among endoscopists. This national registry included centers all over the country, including both university and community hospitals, representing a reliable snapshot of Italian endoscopic facilities.

Indications for biliary drainage in dMBS include relief of jaundice in unresectable patients before neoadjuvant therapy and preoperative biliary drainage 24 . As expected, in our registry, the most common indication for biliary drainage was jaundice palliation in unresectable patients (60%). Preoperative biliary drainage should be reserved for selected cases. In our registry, among the 177 patients undergoing preoperative biliary drainage, 71% had severe jaundice (bilirubin levels > 10 mg/dL), and biliary drainage can be considered a correct indication. Among the other 29% of patients, the indication for preoperative biliary drainage was delayed surgery; however, 12% of patients underwent surgery within 7 days from ERCP. In this patient subgroup, biliary drainage may be considered an overtreatment leading to increased costs. Nevertheless, ERCP can be burdened by AEs (e.g. post-ERCP acute pancreatitis) that may further delay or even preclude surgery; thus, ERCP should be performed only when strictly indicated.

Another critical point underscored by our registry is excessive use of PSs in preoperative biliary drainage (42%), which may be attributed to the following two explanations: 1) PSs may be used in this setting to reduce costs; and 2) surgeons may prefer PSs over SEMSs. However, neither explanation is evidence-based. In a meta-analysis of five studies (one RCT and four non-RCTs) including 704 patients, SEMSs presented a considerably lower need for reintervention after preoperative biliary drainage as compared with PS (3.4% vs. 14.8%, *P* < 0.0001) 14 .

Consequently, PSs are not associated with lower costs, considering the increased need for reintervention. Moreover, the postoperative pancreatic fistula rate was significantly lower for SEMSs than for PSs (5.1% vs 11.8%, *P* = 0.04), with no differences in surgical complications or mortality rates 14 . Moreover, in the setting of palliative drainage, excessive use of PSs was registered (15%). This may be associated with local facilities, because SEMSs are more expensive and may not be available in every center. To the contrary, PSs may be preferred when the pathological diagnosis is not yet available; however, an FC-SEMS should be the stent of choice in this setting. In fact, FC-SEMSs have longer patency as compared with PSs and can be easily removed if pathological results exclude malignancy 25 . Interestingly, PSs were more commonly used in university hospitals, patients with biliary tract cancers, locally advanced and resectable cancers, patients who were candidate for chemotherapy, and patients without a histological diagnosis ( Fig. 2 ). The association between university hospitals and PS placement is unclear. It may depend on the local facility of the specific university hospitals included and it may represent a selection bias of the study.

ESGE recommends use of U-SEMS only in cases with a pathologically confirmed diagnosis of malignancy 13 . In our registry, 55% of patients receiving U-SEMSs did not have a pathologically confirmed diagnosis at time of stent placement. A previous study reported that 5% to 10% of patients undergoing surgery for pancreatic cancer may have a final benign diagnosis 26 27 . Therefore, even if clinical and radiological assessments seem to be unequivocal for pancreatic cancer, the histological diagnosis should be obtained before U-SEMSs placement because they cannot be removed due to tissue ingrowth 8 ; therefore, PC-SEMSs and FC-SEMSs have been designed to allow stent retrieval in order to perform a temporary treatment 9 11 28 , particularly if the final histology is not available. The advantages of FC-SEMSs compared with PSs are the larger diameter, need for fewer stent exchanges, lower risk of stent occlusion, and longer patency 29 . The main limitation of use of FC-SEMSs are their high risk of migration, ranging from 20% to 40% 12 30 . Therefore, FC-SEMSs with antimigratory mechanisms, including flared ends, anchoring fins, and anchoring flaps, have been designed to reduce migration risk 31 . In our study, U-SEMSs without a pathologically confirmed diagnosis were implanted in patients with clearly metastatic disease on cross-sectional imaging and advanced age or poor general conditions precluding any surgical approach; final histology confirmed a malignant disease in all cases. The endoscopists preferred U-SEMSs in these cases due to reduced risk of stent migration and cholecystitis. However, today, owing to availability of FC-SEMSs with antimigratory mechanisms, use of U-SEMSs in patients without a pathological confirmation of malignancy is no longer justified.

Endoscopic stenting for biliary drainage should be considered a temporary treatment; thus, stent occlusion occurs with every type of biliary stent. PSs have shown shorter patency time when compared with SEMSs, and the occlusion mechanism is generally due to biliary sludge formation inside the plastic stent 17 . SEMSs generally have longer patency (6 months) 17 18 . U-SEMSs usually get occluded because of tissue ingrowth through the metal mesh, whereas FC-SEMSs get occluded due to sludge formation inside the stent 16 . In our registry, among the 75 patients receiving a second ERCP for stent occlusion, 83% had treatment consistent with the ESGE guidelines. However, 26% of patients with an occluded plastic stent received a new plastic stent, which is against the current ESGE guidelines, perhaps because of local facilities and device availability.

Although ERCP is a minimally invasive procedure, it is burdened by risk of AEs. In our study, 10.7% of patients experienced early AEs. Incidence of post-ERCP acute pancreatitis in our registry (4.6%) is in line with the current literature 32 . Eighteen RCTs evaluated the role of rectal nonsteroidal inflammatory drugs (NSAIDs) in prevention of post-ERCP pancreatitis and showed that rectal NSAIDs were associated with a significant reduction in odds of post-ERCP acute pancreatitis (odds ratio, 0.49; 95% confidence interval, 0.37–0.65; I ^2^ 38.6%) 33 . Our registry shows good adherence of Italian centers to this guideline, because 94.1% of endoscopy units routinely administered 100 mg of rectal indomethacin right before ERCP. In our registry, post-sphincterotomy bleeding occurred in 3% of patients. Current ESGE guidelines recommend against routine endoscopic biliary sphincterotomy before inserting a single stent to reduce bleeding 13 . Despite this recommendation, in our study, biliary sphincterotomy was performed in 87.9% of patients. Sphincterotomy is generally performed to increase the working space through the papilla, allowing insertion of devices. Moreover, biliary sphincterotomy reduces occurrence of post-ERCP acute pancreatitis 34 . However, a meta-analysis of 17 studies (five RCTs and 12 observational studies) involving 2,710 patients reported no significant difference in risk of post-ERCP pancreatitis between patients with and without endoscopic sphincterotomy when biliary stenting was performed ( *P* = 0.01) 35 . Infective complications, including cholangitis and cholecystitis, are uncommon post-ERCP complications and the ESGE guidelines recommend against routine administration of antibiotic prophylaxis before ERCP. However, in our registry, antibiotic prophylaxis was reported by 70.6% of centers. In the era of antibiotic resistance, use of antibiotic prophylaxis should be carefully reevaluated.

## Conclusions

The present investigation is a large prospective study that evaluated endoscopic management of dMBS in 17 centers in Italy, documenting a reliable snapshot of Italian endoscopic practice. Our results showed that ERCP is highly effective in achieving biliary drainage in dMBS (success rate 97.8%) with a relatively low risk of AEs. The advantages of the present study include its prospective design and involvement of endoscopic centers from different areas and hospital settings, which ensured a reliable patient sample and large sample size. Its main limitations are possible selection bias, because not all centers performing ERCP in Italy participated in the study, and lack of complete follow-up in all the patients. Moreover, this is a national study, and although it provides a wide geographic distribution of enrolling centers, its generalizability to the European population needs to be confirmed. Our study findings may be helpful to improve use of biliary stents in dMBS management because they inform scientific societies of digestive endoscopy about lack of adherence of endoscopists to guidelines on some specific topics.

## Supplementary Material

Supplementary Material Zusatzmaterial
