# Supplementary material for: Adherence to ESGE guidelines on biliary stenting in malignant distal strictures: Results from a prospective Italian registry
Source: Endosc Int Open. 2026 Jan 26;14:a27779199. doi: 10.1055/a-2777-9199 (PMC12908914; doi:10.1055/a-2777-9199)
Supplement: Supplementary file 1 — Supplementary Material [file 10-1055-a-2777-9199_27791268.pdf]

Supplementary table 1 Procedure characteristics.

**Procedure details**	**N (%)**
Histological diagnosis available at time of ERCP (missing data n = 26)	
Yes	284 (34.7)
No	535 (65.3)
Sphincterotomy performed (missing data n = 6)	739 (88.1)
Type of biliary stent	
Plastic stent	189 (22.4)
Metallic stent	656 (77.6)
UC-SEMS	179 (21.2)
PC-SEMS	62 (7.3)
FC-SEMS	415 (49.1)
**Plastic stent characteristics**	
Diameter (missing data n= 4)	
Ø 7F	12 (6.5)
Ø 8.5F	11 (6)
Ø 10F	149 (80.4)
Ø 11.5F	13 (7)
Length	
5 cm	44 (23.3)
7 cm	56 (29.6)
9 cm	78 (41.3)
12 cm	11 (5.8)
**SEMS characteristics**	
Diameter (missing data n=3)	
Ø 6-8 mm	23 (3.5)
Ø 10 mm	630 (96)
Length (missing data n=1)	
4 cm	245 (37.4)
6 cm	363 (55.4)
8 cm	47 (7.2)
Cystic duct infiltration (missing data n = 31)	
No	492 (60.4)

Yes	56 (6.9)
Duct not opacified on cholangiography	266 (32.7)
Cystic duct covered by metal stent (missing data n = 27)	
No	386 (47.2)
Yes	162 (19.8)
Duct not opacified on cholangiography	270 (33)
Aim of the biliary drainage	
Preoperative	131 (15.5)
Neoadjuvant chemoradiotherapy	207 (24.5)
Palliative care	507 (60)
Surgery	180 (21.3)
Curative intent	
Duodenopancreatectomy	148 (82.4)
Left/distal pancreatectomy	17 (8.3)
Exploratory laparotomy	18 (9.3)

ERCP, endoscopic retrograde cholangiopancreatography; FC-SEMS, fully covered self-expandable metal stent; PC-SEMS, partially-covered self-expandable metal stent; U-SEMS, uncovered self-expandable metal stent.

Supplementary table 2 Characteristics of patients stratified according to geographical area in Italy.

Variable	Northern (n = 513)	Central (n = 224)	Southern (n = 108)	*P* value
**Patient details**				
Age (years, mean) (SD)	70 (11)	71 (12)	73 (11)	NS
	N (%)	N (%)	N (%)	
Gender (male)	269 (52.9)	114 (51.1)	50 (46.3)	NS
Malignant obstruction				
Pancreatic cancer	403 (78.6)	192 (85.7)	69 (63.9)	NS
Distal cholangiocarcinoma	49 (9.6)	21 (9.4)	22 (20.4)	NS
Ampullary cancer	31 (6)	4 (1.3)	14 (13)	NS
Metastases	20 (3.9)	4 (1.8)	2 (1.8)	NS
Other	10 (1.9)	4 (1.8)	1 (0.9)	NS
Disease stage				
Resectable	162 (31.9)	73 (33)	26 (24.8)	NS
Locally advanced	203 (40)	89 (40.3)	56 (53.3)	NS
Metastatic disease	143 (29.1)	59 (26.7)	23 (21.9)	NS
**Procedure details**				
Histological diagnosis available at time of ERCP	309 (60.2)	148 (66.1)	78 (72.2)	< 0.0001
Sphincterotomy performed	439 (85.6)	203 (90.6)	97 (89.8)	NS
The type of biliary stent				
Plastic	102 (19.9)	60 (26.8)	27 (25)	NS
Metallic	411 (80.1)	164 (73.2)	81 (75)	< 0.0001
U-SEMS	52 (10.1)	117 (52.2)	10 (9.3)	
PC-SEMS	37 (7.2)	17 (7.6)	8 (7.4)	
FC-SEMS	332 (62.8)	30 (13.4)	63 (58.3)	
Aim of biliary drainage				
Preoperative	107 (20.9)	51 (22.8)	21 (19.8)	NS
Neoadjuvant chemoradiotherapy	117 (22.8)	65 (29.2)	14 (13.2)	NS

Palliative care	289 (56.3)	107 (48)	71 (67)	NS
Patient candidate for surgery	134 (26.2)	58 (25.9)	13 (12.4)	NS

FC-SEMS, fully covered self-expandable metal stent; PC-SEMS, partially-covered self-expandable metal stent; U-SEMS, uncovered self-expandable metal stent.

Supplementary t\Table 3 Variables associated with use of plastic stents in multivariate analysis.

Variable	Plastic stent (n = 189)	Metallic stent (n = 656)	*P* value	Multivariate analysis #1 OR (95% CI)	*P* value	Multivariate analysis #2	
Age (years) (SD)	72 (65-79)	71 (62-79)	0.453	1.001 (0.985-1.018)	0.879	1.003 (0.986-1.020)	0.738
Gender (male)	98 (51.9%)	335 (51.1%)	0.924	0.991 (0.687-1.429)	0.962	1.022 (0.708-1.475)	0.908
Geographic area			0.092				
North (ref.)	102 (54%)	411 (62.6%)		1		1	
Centre	60 (31.7%)	164 (25%)		1.308 (0.858-1.992)	0.212	1.287 (0.842-1.970)	0.243
**South**	**27 (14.3%)**	**81 (12.4%)**		**2.046 (1.109-3.7 77)**	**0.022**	**2.237 (1.205-4.156)**	**0.011**
**University hospital**	126 (26.8%)	344 (73.2%)	**0.001**	**2.132 (1.343-3.386)**	**0.001**	**2.255 (1.412-3.602)**	**0.001**
**Cause of malignant obstruction**			**< 0.0001**				
**Pancreatic cancer (ref.)**	**123 (65.1%)**	**541 (82.5%)**		**1**		**1**	
**Biliary tract cancer**	**53 (28%)**	**87 (13.2%)**		**2.652 (1.649-4.264)**	**< 0.0001**	**2.904 (1.787-4.718)**	**< 0.001**
**Metastases**	**13 (6.9%)**	**28 (4.3%)**		**2.884 (1.343-3.386)**	**0.001**	**3.396 (1.522-7.575)**	**0.003**
Disease stage			< 0.0001				
Resectable (ref.)	84 (45.1%)	177 (27.3%)		1		/	
Locally advanced	58 (31.2%)	290 (44.8%)		0.609 (0.371-1)	0.050	/	
Metastatic disease	44 (23.7%)	181 (27.9%)		0.678 (0.391-1.175)	0.166	/	
Gallbladder stones	37 (19.6%)	97 (14.8%)	0.112	1.080 (0.829-1.407)	0.570	1.321 (0.817-2.134)	0.256
Serum bilirubin (mg/dL)	12.2 (8.2-16.3)	12 (7.5-17.4)	0.937	0.995 (0.969-1.023)	0.738	1.086 (0.833-1.416)	0.543
**Histological diagnosis available**	**31 (16.7%)**	**253 (40%)**	**< 0.0001**	**0.319 (0.203-0.503)**	**< 0.001**	**0.321 (0.203-0.506)**	**< 0.001**

**Aim of biliary stenting**			< 0.0001				
**Preoperative (ref.)**	**53 (28%)**	**78 (11.9%)**		/		1	
**Neoadjuvant chemoradiotherapy**	**48 (25.4%)**	**159 (24.2%)**		**/**		**0.190 (0.075-0.482)**	**< 0.001**
**Palliative care**	**88 (46.6%)**	**419 (63.9%)**		**/**		**0.364 (0.164-0.808)**	**0.013**
Did the patient undergo surgery?	67 (35.5%)	133 (20.3%)	< 0.0001	1.579 (0.9.56-2.607)	0.074	0.617 (0.274-1.387)	0.243

CI, confidence interval; SD, standard deviation.

Supplementary table 4 Adherence of Italian endoscopy centers to ESGE guidelines with stratification according to geographic area.

Variable	Northern	Central	Southern	*P* value
Rate of pts with a clear indication for preoperative BD	80 (92%)	31 (89%)	7 (88%)	0.671
Rate of patients with jaundice and candidates to neoadjuvant chemotherapy who underwent BD	116 (100%)	62 (97%)	15 (100%)	0.262
Rate of patients in whom SEMS was used for preoperative BD	65 (75%)	10 (29%)	2 (25%)	**< 0.001**
Rate of patients in whom a SEMS of 10 mm was used for preoperative BD	64 (98%)	9 (90%)	2 (100%)	0.289
Rate of patients in whom SEMS was used in the setting of neoadjuvant chemotherapy	85 (70%)	61 (87%)	13 (81%)	**0.023**
Rate of patients in whom SEMS was used in the setting of palliative BD	260 (85%)	93 (78%)	66 (79%)	0.110
Rate of pts in whom a U-SEMS was placed before histological confirmation of the dMBO aetiology	17 (33%)	61 (52%)	1 (12.5%)	**0.011**
Rate of pts in whom a sphincterotomy was not performed routinely	69 (14%)	21 (9%)	10 (9%)	0.183

BD, biliary drainage; dMBO, distal malignant biliary obstruction; ESGE, European Society of Gastrointestinal Endoscopy; SEMS, self-expandable metal stent.

Thieme

Supplementary table 5 Adherence to ESGE guidelines according to hospital setting.

Variable	University hospital	Community hospital	*P* value
Rate of patients who had clear indication for preoperative BD	65 (85%)	53 (98%)	0.014
Rate of patients with jaundice and candidates for neoadjuvant chemotherapy who underwent BD	129 (98%)	65 (100%)	1
Rate of patients in whom SEMS was used for preoperative BD	46 (61%)	31 (57%)	0.856
Rate of patients in whom a 10-mm SEMS was used for preoperative BD	44 (96%)	31 (100%)	0.239
Rate of patients in whom SEMS was used in the setting of neoadjuvant chemotherapy	101 (73%)	58 (84%)	0.115
Rate of patients in whom SEMS was used in the setting of palliative BD	197 (77%)	222 (88%)	0.110
Rate of patients in whom a U-SEMS was placed after histological confirmation of dMBO etiology	69 (51%)	10 (23%)	**0.001**
Rate of patients in whom a sphincterotomy was not performed routinely	44 (9%)	56 (15%)	**0.011**

BD, biliary drainage; dMBO, distal malignant biliary obstruction; ESGE, European Society of Gastrointestinal Endoscopy; SEMS, self-expandable metal stent; U-SEMS, uncovered self-expandable metal stent.
